# Metabolomic Signatures in Adults with Metabolic Syndrome Indicate Preclinical Disruptions in Pathways Associated with High-Density Lipoprotein Cholesterol, Sugar Alcohols

**DOI:** 10.21203/rs.3.rs-5989567/v1

**Published:** 2025-02-14

**Authors:** K. A. Lewis, Benjamin M. Stroebel, Alka M. Kanaya, Bradley Aouizerat, Kayla D. Longoria, Elena Flowers

**Affiliations:** University of California, San Francisco; University of California, San Francisco; University of California, San Francisco; New York University; University of California, San Francisco; University of California, San Francisco

**Keywords:** metabolic signatures, type 2 diabetes risk, prediabetes, branched chain amino acids, high density lipoprotein cholesterol, cardiovascular risk, triglycerides, metabolic syndrome, metabolomics, sugar alcohols

## Abstract

**Background:**

Metabolic syndrome is a pressing public health issue and risk factor for the development of type 2 diabetes (T2D) and cardiovascular disease (CVD), yet clinical practice is lacking in biomarkers that represent pre-clinical perturbations of the heterogenous subtypes of risk. This study aimed to characterize the baseline metabolome in relation to known clinical characteristics of risk in a sample of obese adults.

**Methods:**

Untargeted metabolome data from *N* = 126 plasma samples with baseline data from a previously completed study including obese adults with metabolic syndrome. Metabolites were acquired using validated liquid chromatography mass spectrometry methods with 15–25 internal standards quantified by peak heights. Pearson’s correlations were used to determine relationships between baseline metabolites, sample characteristics (e.g., age, body mass index (BMI)), and atherosclerotic clinical characteristics (e.g., high-density lipoprotein cholesterol (HDL), low-density lipoprotein cholesterol (LDL), triglycerides), adjusting for multiple comparisons using the Benjamini-Hochberg False Discovery Rate (FDR) method. Differences in metabolite levels between clinical classifications of dysglycemia (e.g., normal, prediabetes, diabetes) at baseline were assessed using ANOVA and adjusted for multiple comparisons and adjusted for covariates.

**Results:**

The sample consisted primarily of female (74%) participants, predominantly white (70%), with an average age of 56 years. After FDR adjustment, two baseline metabolites were significantly associated with age (xylose, threitol), two with BMI (shikimic acid, propane-1,3-diol), one with LDL (tocopherol-alpha), and 42 with HDL cholesterol. Three metabolites were significantly associated with fasting blood glucose (FBG) levels at baseline (glucose, gluconic acid lactone, pelargonic acid).

**Conclusions:**

This study identified novel metabolite associations with known markers of T2D and CVD risk. Specific metabolites, such as alpha-tocopherol, branched-chain amino acids (BCAAs), and sugar-derived metabolites like mannose and xylose, were significantly associated with age, BMI, lipid profiles, and glucose measures. Although most sample participants had normal HDL cholesterol at baseline, 42 metabolites including branched chain amino acids were significantly associated with HDL, suggesting pre-clinical perturbations in biological pathways associated with both diabetes and cardiovascular comorbidities. Metabolomic signatures Specific to prediabetes and metabolic syndrome can enhance risk stratification and enable targeted prevention strategies for T2D. Longitudinal studies are needed to understand how these associations change over time in at-risk individuals compared with controls.

## Introduction

Type 2 diabetes (T2D) and cardiovascular disease (CVD) represent significant global public health burdens, with T2D affecting over 422 million people and CVD being the leading cause of death globally, accounting for 17.9 million deaths each year (1–2). Metabolic syndrome, a major contributor to both T2D and CVD, affects approximately one-third of adults in the United States and has similar prevalence rates globally (3). Metabolic syndrome is characterized by a cluster of risk factors including elevated fasting blood glucose (FBG) levels, elevated waist circumference, elevated blood pressure, high triglyceride levels, and low high-density lipoprotein (HDL) cholesterol levels. While recognized as not being greater than the sum of its parts, the component risk factors of this syndrome, and particularly in combination, significantly increase the risk of developing both T2D and CVD and related health problems (4).

Understanding the precise biological mechanisms underlying the progression from metabolic syndrome to T2D and CVD is crucial for prevention, early detection, and intervention. Despite advancements, our understanding of metabolic biomarkers and pathways in metabolic syndrome remains incomplete due to several key gaps. The multiple individual risk factors that comprise metabolic syndrome each potentially have both shared and discrete mechanisms, which current biomarkers like HbA1c and fasting glucose in isolation fail to adequately capture (4). Further, these conventional biomarkers often identify metabolic syndrome at a stage when significant metabolic alterations have already occurred, failing to detect early, subtle changes that could allow for more timely interventions and prevention of long-term complications (5–7). Additionally, the heterogeneity in metabolic syndrome is not well represented by existing biomarkers, limiting their diagnostic and predictive accuracy (8–9).

Metabolomics offers a solution to these gaps by enabling the detailed analysis of the full spectrum of metabolites, providing novel biomarkers that may offer broader insights into changes in biochemical pathways for early detection of risk (10–12). Studies have demonstrated the potential for metabolomics to identify Specific metabolic signatures and pathways associated with the progression of metabolic syndrome to chronic cardiometabolic disease, underscoring the necessity for further research in this area (5–7, 13).

Across previous metabolomics studies, there is accumulating evidence for associations between individual metabolites and metabolic syndrome and related outcomes. Branched-chain amino acids (BCAAs) such as valine, leucine, and isoleucine were described as potential predictors of insulin resistance and T2D risk, alongside aromatic amino acids like phenylalanine and tyrosine (6, 14–15). Similarly, uric acid has been described as a potential biomarker for metabolic changes associated with metabolic syndrome (5). Lipid-related metabolites, including lysophosphatidylcholines (lysoPCs) and ceramides, highlighted disruptions in lipid metabolism linked to T2D and CVD risk (16). Additionally, intermediates from the tricarboxylic acid (TCA) cycle, such as succinate and fumarate, underscore the role of mitochondrial dysfunction in early metabolic disturbances (17). Though promising, the dynamic nature of metabolomics makes results difficult to replicate, and so existing research has not yet confirmed how Specific metabolomic signatures can enhance the detection of early metabolic changes linked to T2D and CVD risk, nor how these signatures complement traditional biomarkers like fasting glucose and HbA1c to improve early diagnosis and prevention. The purpose of the present study is to identify metabolomic signatures associated with metabolic syndrome components that increase risk for T2D and CVD in a sample of underactive obese adults. By pinpointing these early metabolic changes, we aim to enhance our understanding of prodromal and individual T2D and CVD pathogenesis and pave the way for more effective preventative interventions.

## Methods

### Parent Study

This study leverages data from the Practicing Restorative Yoga vs. Stretching for the Metabolic Syndrome (PRYSMS) trial conducted by Kanaya et al. (2013), NCT01024816. (18) The study was conducted in concordance with the Declaration of Helsinki and all participants provided informed consent. The PRYSMS trial was a 48-week randomized controlled trial aimed at evaluating the effects of restorative yoga compared to active stretching on metabolic risk factors among underactive adults who met the clinical criteria for metabolic syndrome and were at high risk for T2D. Participants were recruited from the San Francisco and San Diego communities over a three-year period. Blood samples were collected at baseline to assess various metabolic markers, including fFBG, HbA1c, and lipids, and remaining samples were stored frozen in a biobank at −80°F for subsequent analyses. The methods by which the sample and clinical variables were acquired for the parent study have been previously described in detail. (18).

### Current Study

We collected *de novo* metabolomics data from N = 126 participants’ blood samples with at least 200 μL of blood remaining from the original PRYSMS trial in the biobank. Metabolites were analyzed with the previous data collected from the PRYSMS trial, which included baseline sample characteristics (i.e., age, gender, race and ethnicity) and clinical characteristics (i.e., body mass index (BMI), FBG, fasting high density lipoprotein (HDL) cholesterol, fasting triglycerides, and low-density lipoprotein (LDL) cholesterol).

In addition, we compared differences in metabolite levels based on the American Diabetes Association’s guidelines to define normal, prediabetes, and diabetes categories and normal and abnormal HDL cholesterol levels (19). FBG levels were defined as normal if they were < 100 mg/dL, as prediabetes if between 100–125 mg/dL, and as diabetes if ≥ 126 mg/dL (19). For HgbA1c, the ADA defines normal as ≤ 5.6%, prediabetes between 5.7–6.4%, and diabetes as ≥ 6.5% (19). HDL was defined as normal or abnormal based on gender-dependent guidelines: HDL levels for females were denoted as abnormal if less than 50 mg/dL, while levels for males were abnormal if < 40 mg/dL (19).

### Plasma Sample Processing and Metabolite Data Acquisition

#### Instruments

The University of California at Davis (UC Davis) Metabolomics Core and Research Laboratories at the UC Davis Genome Center in Davis, CA utilized a Gerstel CIS4 with dual MPS injector, an Agilent 6890 GC, and a Pegasus III TOF MS for the metabolomics analysis.

#### Injector Conditions

The Agilent 6890 GC was equipped with a Gerstel automatic liner exchange system (ALEX) that included a multipurpose sample (MPS2) dual rail and a Gerstel CIS cold injection system (Gerstel, Muehlheim, Germany). The temperature program for the CIS system was set from 50°C to a final temperature of 275°C at a rate of 12°C/s, with a hold time of 3 minutes. An injection volume of 0.5 μl was used, with an injection speed of 10 μl/s on a splitless injector with a purge time of 25 seconds. The liner (Gerstel #011711–010-00) was changed after every 10 samples using the Maestro1 Gerstel software vs. 1.1.4.18. The 10 μl injection syringe was washed three times with 10 μl ethyl acetate before and after each injection.

#### Gas Chromatography Conditions

A 30 m long, 0.25 mm i.d. Rtx-5Sil MS column with a 0.25 μm 95% dimethyl 5% diphenyl polysiloxane film and an additional 10 m integrated guard column (Restek, Bellefonte PA) was employed. Helium (99.9999% pure) with a built-in purifier (Airgas, Radnor PA) was used as the carrier gas at a constant flow rate of 1 ml/min. The oven temperature was held constant at 50°C for 1 minute, then ramped at 20°C/min to 330°C, and maintained at 330°C for 5 minutes.

#### Mass Spectrometer Settings

The Leco Pegasus IV time of flight mass spectrometer was controlled using the Leco ChromaTOF software vs. 2.32 (St. Joseph, MI). The transfer line temperature between the gas chromatograph and mass spectrometer was set to 280°C. Electron impact ionization at 70V was employed, with an ion source temperature of 250°C. The acquisition rate was set at 17 spectra/second, with a scan mass range of 85–500 Da.

### Data Analysis

Metabolites were log transformed and converted to z scores prior to analysis. Pearson’s Correlations were used to determine relationships between baseline metabolites, sample characteristics (i.e., age, BMI), and the clinical characteristics (i.e., HDL and triglycerides), adjusting for multiple comparisons using the Benjamini Hochberg False Discovery Rate (FDR) method (*q* < 0.2). (20) The pathway analysis was conducted using MetaboAnalyst 6.0 (21) to identify the enriched Kyoto Encyclopedia of Genes and Genomes pathways (22).

Differences in metabolite levels between clinical classifications (i.e., normal, prediabetes, and diabetes) of dysglycemica (i.e., FBG, HbA1c) at baseline were assessed using analysis of variance (ANOVA) and then adjusted for multiple comparisons using FDR. Differences for normal versus abnormal HDL were assessed using independent samples t-tests and adjusted for multiple comparisons using FDR.

Significant associations after FDR adjustment were further adjusted for covariates of age, BMI, and lipid-lowering medications (e.g., statins) using multiple linear regression. Sensitivity analyses were conducted to compare waist circumference with BMI as a body composition measure, as well as comparing the calculated TG/HDL ratio to evaluate if it provided additional associations beyond those of the individual predictors.

## Results

Sample and clinical characteristics of the participants included in the current study are presented in Table 1. The overall sample was mostly female, white race, 56 ± 6 years old, obese, had prediabetes and dyslipidemia, and normotensive. There were 31 participants (25%) taking a statin or lipid lowering medication.

**Figure 1:**
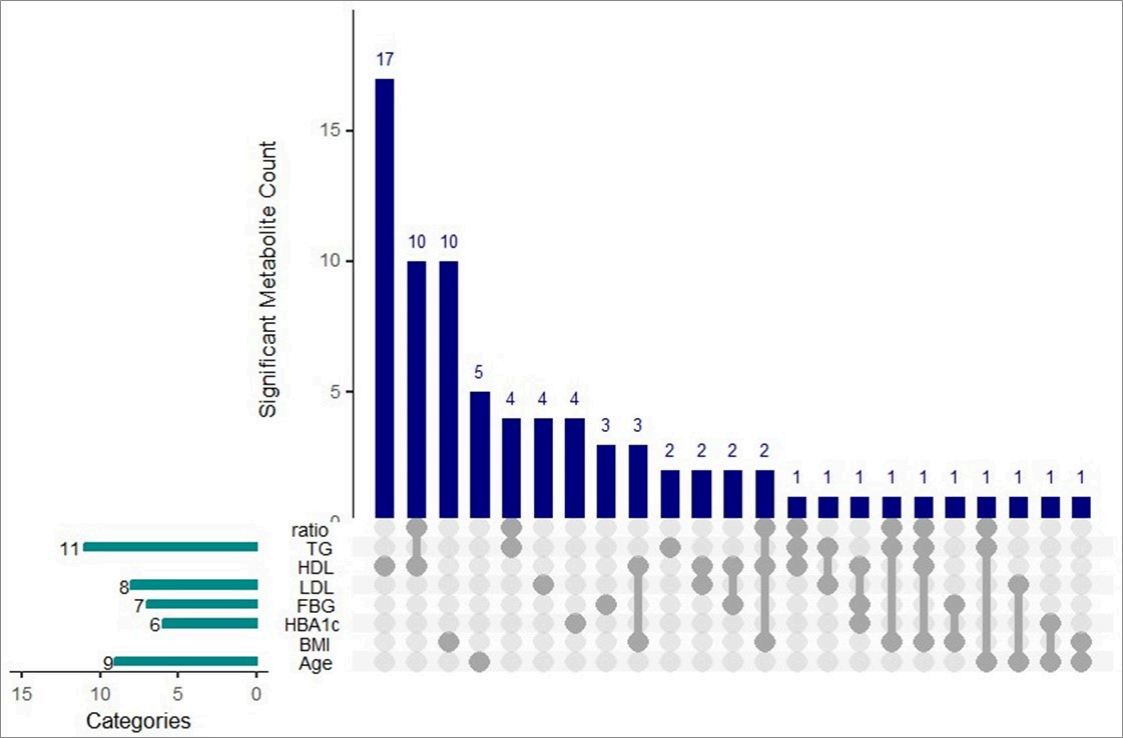
Upset Plot Reflecting Metabolites Significantly Associated with Sample and Clinical Characteristics at Baseline (FDR *q* < 0.2) This Upset plot depicts the significant metabolite count associated with various risk factors. The vertical bars represent the number of significant metabolites for each Specific combination of risk factors. The horizontal bars on the left indicate the number of significant metabolites within each individual risk factor. Categories include TG/HDL ratio, TG (triglycerides), HDL (high-density lipoprotein), LDL (low-density lipoprotein), FBG (fasting blood glucose), HbA1c, BMI (body mass index), and age. The connected dots below the vertical bars highlight the intersections of these risk factors.

**Figure 2:**
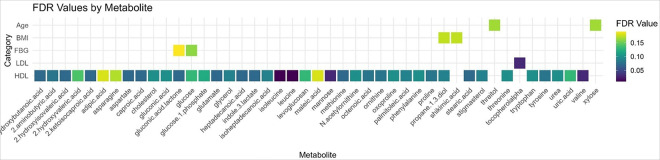
Correlation Plot of Significant Associations (p < 0.5) Between Age, Body Mass Index (BMI), Fasting Blood Glucose (FBG), Low Density Lipoprotein (LDL) Cholesterol, and High-Density Lipoprotein (HDL) Cholesterol at Baseline

**Figure 3:**
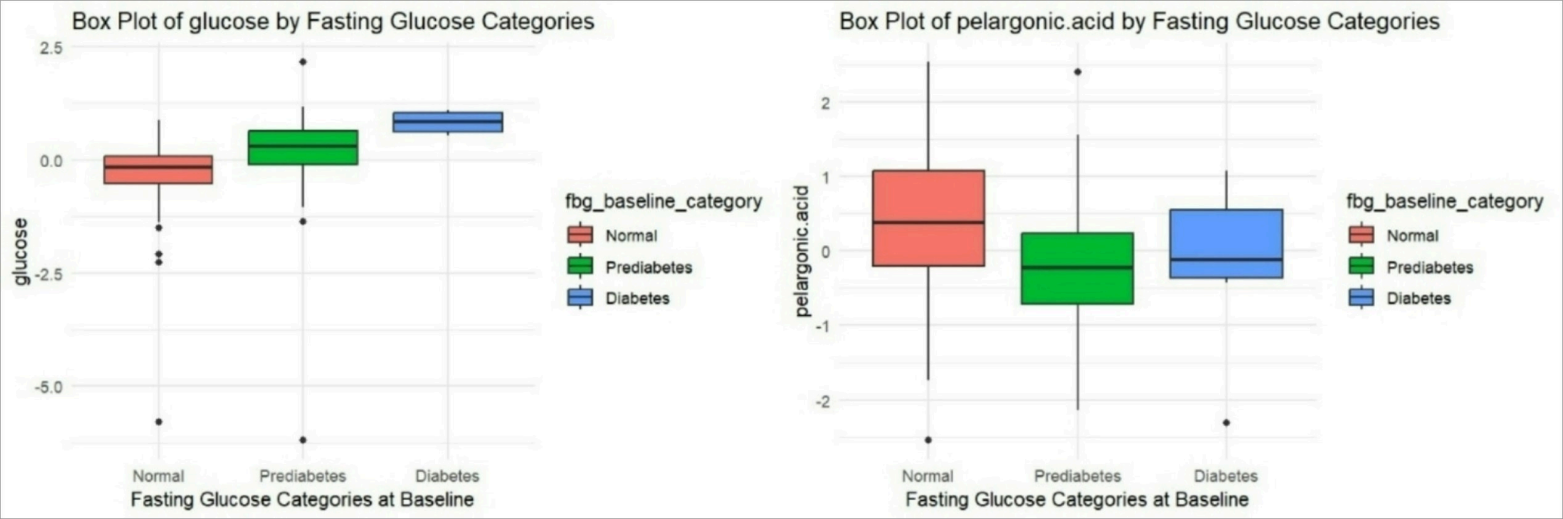
Box Plot of Metabolites Significantly Associated with Fasting Blood Glucose at Baseline, *q*< 0.05

**Figure 4:**
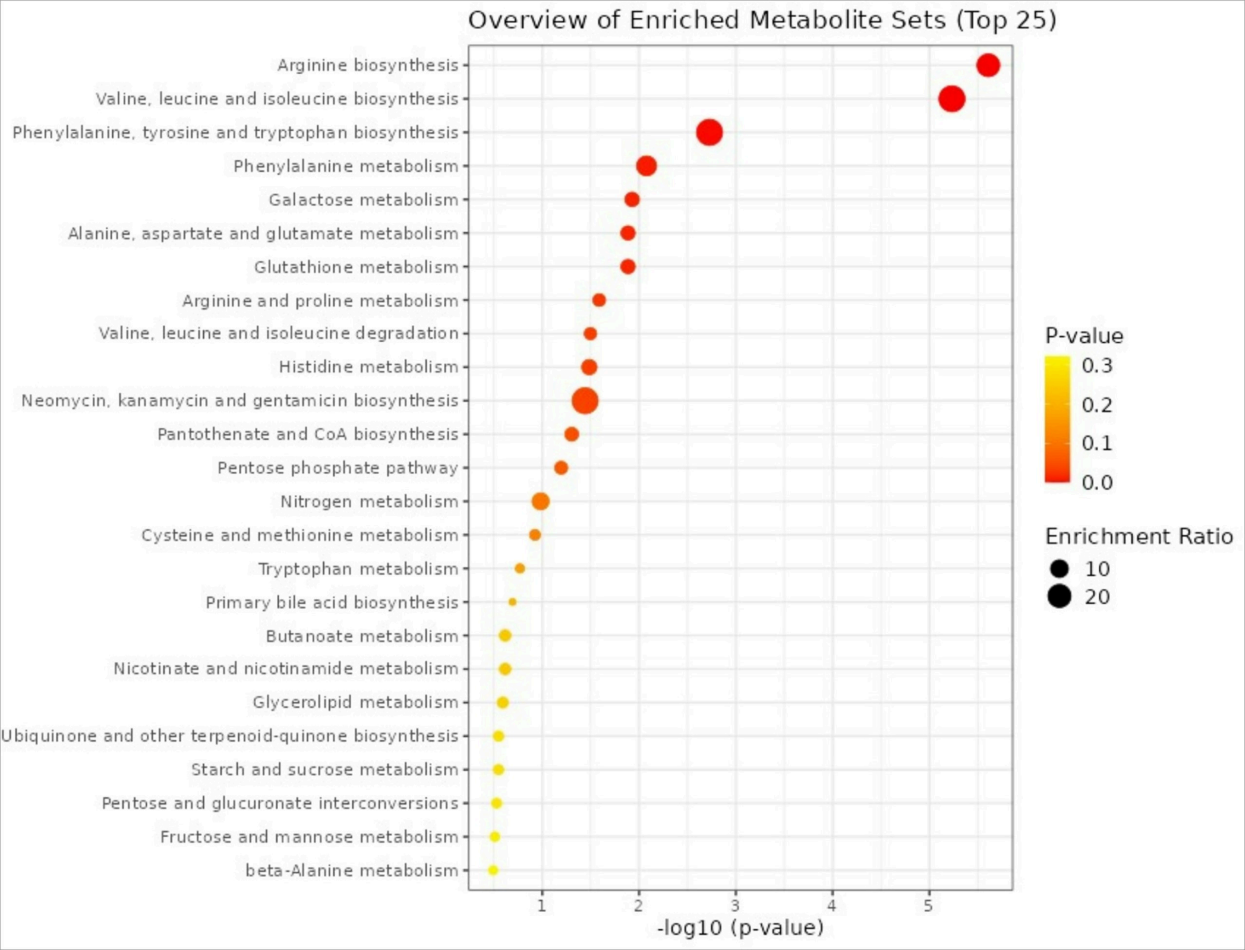
Diagram of Enriched KEGG Pathways from Baseline Metabolites Significantly Associated with One or More Sample or Clinical Characteristic (FDR < 0.2)

Results of the metabolite association analyses are reported in Figs. 1–4 and **Supplementary Tables 1–2**.

After adjustment for multiple comparisons, 48 metabolites were significantly associated with sample or clinical characteristics (q < 0.2). Xylose and threitol were significantly associated with age, shikimic acid and propane-1,3-diol were associated with BMI, glucose metabolite and gluconic acid lactone were significantly associated with baseline continuous FBG levels, and an additional 42 metabolites were associated with HDL cholesterol (Figure 2). Propane-1,3-diol was associated with both HDL and BMI after adjustment for multiple comparisons (q < 0.2). Using the more stringent cutoff of q < 0.05, valine, leucine, isoleucine, and mannose were associated with HDL, and alpha-tocopherol was associated with LDL cholesterol.

Glucose measured by the metabolomic assay and pelargonic acid were significantly associated with FBG categories (i.e., normal, prediabetes, and diabetes) at baseline (Figure 3, **Supplementary Files 3–4**). No metabolites were significantly different between categories of HbA1c, HDL, or TG after adjustment for multiple comparisons. The ratio of TG/HDL was not associated with any metabolites after adjustment for multiple comparisons.

Figure 4 depicts the top biologic pathways represented by the significant metabolites. MetaboAnalyst yielded arginine biosynthesis, valine, leucine, and isoleucine biosynthesis, and phenylalanine, tyrosine and tryptophan biosynthesis as the top three significantly enriched pathways (q < 0.05).

Multiple linear regression was used to adjust for covariates of the significant associations, and the results are presented in Supplementary File 5. All models remained significant after adjusting for covariates except for propane-1,3-diol and FBG.

## Discussion

This study identified several novel metabolite associations with the components of the metabolic syndrome that increase risk for T2D and CVD. Metabolites were associated differently with age, BMI, FBG, and lipid measures, indicating potentially disparate mechanisms involved and supporting the heterogenous nature of T2D risk. Despite most participants having normal HDL levels based on current ADA guidelines (19), the metabolic signature of risk in this sample of participants was most strongly associated with HDL levels and metabolites from the branched chain amino acids, sugar-derived metabolites (i.e., glucose, mannose, xylose, threitol), and environmental chemicals, food additives or preservatives (gluconic acid lactone, propane-1,3-diol, pelargonic acid, alpha tocopherol). These findings may point to early, preclinical metabolic disruptions associated with risk for T2D and cardiovascular comorbidities.

### Metabolic Signatures and Age

We found that xylose and threitol were both significantly positively associated with age in our sample of participants, and the model remained significant after adjusting for covariates. Xylose is a direct metabolite of the natural sugar alcohol xylitol (23). In T2D, xylose can act as an inhibitor of sucrase, and supplementation has been associated with improved FBG in humans and mouse models and improved lipid profiles in mouse models (23–24). A seminal paper found that as humans age, they are less effective at eliminating circulating xylose from the body via the urine (25). This decreased elimination may explain the higher levels of circulating xylose in participants of an older age in our sample.

Less is known about the impact of higher circulating levels related to aging because few studies have been conducted in humans. A study assessing the transcriptome and proteome of kidney tissue samples in swine on a xylose diet found that the differentially expressed genes were related to lipid biosynthesis and fatty acid metabolism (26). In our regression model, lipid-lowering medications were significant independent predictors for this sample. Subsequent studies that characterize the metabolic characteristics of older participants in relation to their circulating xylose levels may yield useful information about the utility and risks of xylose supplementation in high-risk individuals.

The sugar alcohol threitol is the end-product of xylose metabolism (27). Altered serum urine levels of threitol have been associated with T2D and altered carbohydrate metabolism (27–28). In mouse models comparing C57– 5’AMP-activated protein kinase (AMPK) knockout mice with a control group, levels of both xylose and threitol were lower in the C57-AMPK knockout mice (28). Threitol is associated with the galactose metabolism pathway, which was in the top 5 of the enriched pathways reflected by our study findings (29). Like xylose, the threitol model was significant after adjusting for covariates, and lipid-lowering medications were a significant, independent predictor in the model.

To the best of our knowledge, this is the first study to directly associate the sugar alcohol threitol with age in humans. Previous work assessing differential microRNA expression in a subset of participants who had completed 2 years of metformin treatment for T2D prevention in the Diabetes Prevention Program found that two differentially expressed microRNAs (i.e., let-7c-5p and miR-130b-3p) associated with the metformin group were also previously linked with antiaging and cellular senescence (30). Future research should explore the associations between sugar alcohol metabolites in older adults and senescence-related microRNA expression.

These sugar alcohol metabolites may reflect age-related metabolic changes in individuals with prediabetes, the nature of which may be influenced by statins or other lipid-lowering medications. However, further research is needed to understand the impact of higher levels of circulating threitol and xylose in relation to aging.

### Body Mass Index (BMI)

Shikimic acid was positively associated with BMI and propane-1,3-diol was inversely associated with BMI in our sample of participants, and both associations remained significant after adjusting for covariates (i.e., age, gender, and lipid-lowering medications). The shikimic acid metabolic pathway leads to the production of aromatic amino acids histidine, phenylalanine, tryptophan, and tyrosine (31–32). Elevated branched chain amino acid (i.e., valine, leucine, and isoleucine) and aromatic amino acid levels in overweight and obese adults are promising biomarkers of prediabetes and early predictors of T2D onset in at risk individuals (33–35). As shikimic acid is upstream in the metabolic pathway that leads to aromatic amino acids, our findings are aligned with previous work that links increased aromatic amino acids in obese individuals. However, the predictive ability of aromatic amino acids for T2D onset may vary by BMI category, gender, or in diverse populations (35). Because our sample was mostly female, our findings suggest that the predictive ability in females may be related to metabolic intermediates instead of the aromatic amino acids themselves.

In contrast, propane-1,3-diol was inversely associated with BMI in our participants and remained significant after adjusting for covariates. This metabolite is a byproduct of microbial fermentation and can be involved in various metabolic processes, including the metabolism of glycerol (36). In cosmetics and pharmaceuticals, 1,3-propanediol can act as a solvent, humectant, and preservative. It helps in maintaining moisture and stability in products (36). To the best of our knowledge, this is the first paper to report an association between propane-1,3-diol and BMI in humans, and the mechanism of association with obesity or risk for type 2 diabetes is unknown.

Elevated shikimic acid in an obese, mostly female population could indicate disruptions in aromatic amino acid metabolism, which has been observed in obesity-related metabolic dysfunctions. The relationships between propane-1,3-diol require further investigation to understand the role it may play in T2D risk.

### Lipoproteins

Alpha-tocopherol, or vitamin E, was positively associated with LDL cholesterol in our sample, and another 42 metabolites were associated with HDL cholesterol. Two of the metabolites associated with HDL were positive associations (i.e., palmitoleic acid, glycerol) and the rest were inverse associations.

The model for alpha-tocopherol was significant after adjusting for covariates. Gender and lipid-lowering medications as covariates were significant, independent predictors of LDL in the model. Females had a significantly higher mean LDL level in our sample (Female *M*=129 mg/dL ± 35, Male *M*=113 mg/dL ± 34, p=0.03). Gender differences in lipid metabolism are influenced by hormonal variations, differences in lipoprotein profiles, and varied responses to diet and lipid-lowering medications (37–38). These differences underscore the importance of considering gender as a factor in metabolomics studies related to lipid metabolism, metabolic signatures of cardiovascular risk, and the effects of interventions like dietary changes or lipid-lowering medications. Gender-associated variations can also impact the bioavailability and metabolism of nutrients like alpha-tocopherol (37).

Four of the metabolites were significantly associated with HDL using an FDR cutoff of 0.5 including mannose, previously linked with maternal and infant adiposity in the perinatal period (39), and three of the branched chain amino acids (i.e., valine, leucine, and isoleucine). The model for mannose was significant after adjusting for covariates, and gender and age were significant independent predictors of HDL in the model. Mannose metabolism pathways have been found to be significantly enriched in individuals with obesity and metabolic syndrome (40). Altered mannose metabolism may be associated with unfavorable lipid profiles like the lower HDL levels observed in our study, and differences based on gender and age should be considered in future studies.

All models for the branched chain amino acids (BCAAs) were also significant after adjusting for covariates, and gender was an independent significant predictor for all three models. Age was also an independent predictor in the valine model. BCAAs have been documented as significant predictors of metabolic syndrome (41). In our study, all participants had metabolic syndrome, and the BCAAs were only significantly associated with the HDL levels and not with the other lipids. This suggests that the BCAAs may be the best predictor of an HDL-Specific phenotype of T2D risk.

Categorization based on current guidelines of normal versus abnormal HDL cholesterol and TG yielded no meaningful differences in metabolite levels, suggesting that the 42 HDL associations noted in the continuous metabolite levels add value for understanding pathways involved in early, preclinical pathogenesis of risk. Similarly, the TG/HDL ratio diluted the strength of the associations between TG or HDL with the metabolites and did not add value to the analysis beyond the associations with the individual metric in this population.

The strong association with HDL cholesterol suggests significant preclinical metabolic alterations potentially impacting early T2D pathogenesis. The inverse relationship with branched-chain amino acids supports previous findings linking these metabolites to insulin resistance and cardiovascular risk. The individual clinical risk factor measurements (i.e., LDL, HDL, TG) were more effective than ratios or categorization of HDL for assessing relationships with the metabolites.

### Glycemic Status

As expected, the glucose metabolite was positively associated with FBG in our study. We also found propane-1,3-diol and pelargonic acid to have significant inverse associations with FBG at baseline. Metabolism of propane-1,3-diol has typically been investigated in gut bacteria’s glycerol metabolism, which is thought to be different from human circulating glycerol metabolism (42). However, Jin et al. (2021) identified propane-1,3-diol in the livers of hamsters and documented an alternative glycerol metabolic pathway characterized by minimal gluconeogenesis, increased ketogenesis, and associations with oxidative metabolism of glycerol through the TCA cycle (42). Circulating glycerol is elevated in starvation, exercise, and diabetes (43). Further research is warranted to understand the source and mechanisms of propane-1,3-diol in circulation and its associations with both FBG and BMI. After adjusting for covariates, the model of the association between propane-1,3-diol and FBG was no longer significant, with BMI as the only significant independent predictor of propane-1,3-diol levels.

Pelargonic acid, also known as nonanoic acid, is a type of medium chain saturated fatty acid, naturally occurring in plants and is also a common environmental pesticide (44–45). In the review article by Sobczak et al. (2019), pelargonic acid levels were decreased in at least one study of patients with T2D, potentially impacting metabolic regulation and contributing to the disease’s progression. (45)

Further, lipid-lowering medications, such as fibrates and statins, may influence the concentrations of plasma free fatty acids (FFAs) (45). These medications are thought to exert part of their beneficial effects by altering the levels of Specific FFAs. For example, in at least one study of metabolic profiling of coronary heart disease, lipid-lowering therapies were associated with lower levels of pelargonic acid after treatment (46).

However, in our sample the regression model was still significant (p=0.005) after adjusting for covariates, and in the model, lipid-lowering medications were not a significant independent predictor of pelargonic acid levels. Age and BMI were significant independent predictors of pelargonic acid levels in the model. This suggests that the results could be further explained by analyzing levels in relation to dietary intake of plants (e.g., fiber intake, servings of fruits and vegetables consumed each day). Future research should assess for relationships between pelargonic acid and plant intake in the diet.

### Metabolic Pathway Disruptions Associated with HDL Cholesterol

The top biologic pathways represented by significant metabolites — arginine biosynthesis, BCAA biosynthesis, and aromatic amino acid biosynthesis — are involved in maintaining metabolic health (47). Disruptions in these pathways can affect HDL cholesterol levels and function, leading to early metabolic disturbances linked to the pathogenesis of T2D and CVD. Understanding these pathway associations can help in identifying early biomarkers and potential therapeutic targets for preventing and managing these diseases.

#### Arginine Biosynthesis:

Arginine is a precursor for nitric oxide (NO) synthesis, which plays a role in vasodilation and maintaining endothelial function (48–50). Proper endothelial function is crucial for preventing atherosclerosis, a key component of CVD. HDL cholesterol is known to enhance endothelial function partly through increased NO production. Disruptions in arginine biosynthesis can impair NO production, leading to endothelial dysfunction, which is an early indicator of both T2D and CVD. Endothelial dysfunction can contribute to insulin resistance and atherosclerosis, underlying both T2D and CVD pathogenesis.

#### Valine, Leucine, and Isoleucine Biosynthesis:

These BCAAs are important for protein synthesis and energy production (51). Elevated levels of BCAAs have been linked to insulin resistance, a precursor to T2D (51). HDL cholesterol is involved in lipid transport and metabolism, which is interconnected with amino acid metabolism. These metabolic disruptions can lower HDL cholesterol levels and increase the risk of T2D and CVD. Elevated BCAAs can also contribute to inflammation and oxidative stress, exacerbating the development of atherosclerosis and diabetes.

#### Phenylalanine, Tyrosine, and Tryptophan Biosynthesis:

These aromatic amino acids are precursors for several important biomolecules, including neurotransmitters and hormones. They also play roles in regulating metabolic processes and inflammatory responses. HDL cholesterol influences these metabolic pathways through its anti-inflammatory and antioxidant properties. Dysregulation of these amino acid pathways can lead to increased oxidative stress and inflammation, both of which are implicated in the pathogenesis of T2D and CVD. For instance, tyrosine and tryptophan metabolites are involved in insulin signaling and vascular health. Disruptions in these pathways can impair insulin sensitivity and promote atherosclerotic changes, contributing to early T2D and CVD development.

### Limitations

Though the sample did have racial and ethnic diversity, it was still predominantly female, white, and high levels of education, which may limit the generalizability of the findings. The study’s cross-sectional design adds value to our understanding of the metabolic signature associated with heterogenous risk characteristics but limits the ability to infer causality. Longitudinal studies are needed to confirm these associations over time. While comprehensive, the untargeted metabolomic approach may miss some metabolites relevant to T2D progression. Findings should be verified with larger samples. Finally, our analysis was limited to the binary gender categorizations in the parent study. Including a more expansive list of gender categories may yield more nuanced results about the gender-associated relationships observed in our study.

### Future Directions

Based on our findings, future research should involve longitudinal studies, mechanistic studies, integration with genomic data, and subgroup analyses in diverse samples. Metabolomics are dynamic and influenced by many factors. As a result, longitudinal studies are needed that track metabolomic changes over time in diverse populations to validate these findings and better understand the progression from prediabetes to T2D. Investigating the underlying biological mechanisms through the pathways in which identified metabolites influence diabetes risk could provide deeper insights and potential therapeutic targets. Combining metabolomic data with genomic and transcriptomic profiles could enhance the precision of diabetes risk prediction and uncover novel biomarkers. Finally, as T2D risk is heterogenous, studies with large enough samples to explore the metabolic signatures of subgroups are needed. Specifically, future research should consider the unique pathophysiology and disease progression for pre- and postmenopausal women given the findings varied by gender. In addition, studies of diverse populations are needed that are inclusive of global populations most vulnerable to developing diabetes due to lack of access to nourishing food and healthcare.

## Conclusion

The study provides valuable insights into the metabolomic alterations associated with prediabetes and metabolic syndrome. The disparate associations by clinical characteristic support the heterogenous nature of T2D risk and further support the presence of distinct phenotypes of risk. Identifying early metabolic signatures can improve diabetes risk stratification and inform targeted prevention strategies. Future research should build on these findings to develop comprehensive models integrating metabolic, genetic, and clinical data for more effective diabetes prevention and management.

## Figures and Tables

**Table 1.** Sample and Clinical Characteristics

	Baseline Values (*N* = 126) n (%) or mean ± SD

**Age (years)**	56 ± 6

**Gender**	

Female	94 (75%)

**Race and Ethnicity**	

Asian and Pacific Islander	15 (12%)

Black	4 (3%)

Latin	16 (13%)

White	89 (71%)

Other	1 (1%)

**Education Level**	
High School	6 (5%)
Some College	32 (25%)
College Graduate	45 (36%)
Postgraduate Degree	43 (34%)

**BMI (kg/m^2^)**	34.1 ± 7.0

**Glucose (mg/dL)**	103 ± 12

**HbA1c (%)**	5.9 ± 0.4

**Total Cholesterol (mg/dL)**	205 ± 36

**Triglycerides (mg/dL)**	173 ± 72

**HDL Cholesterol(mg/dL)**	49 ± 10

**TG/HDL Ratio**	3.8 ± 2.0

**LDL Cholesterol(mg/dL)**	123 ± 33

**Waist Circumference(cm)**	108.0 ± 13.4

**Hip Circumference (cm)**	115.3 ± 12.5

**Average Systolic (mm Hg)**	125 ± 14

**Average diastolic (mm Hg)**	73 ± 10

## Data Availability

O Due to the secondary analysis nature of the current study, the datasets generated and/or analysed during the current study are not publicly available, but datasets will be made available from the corresponding author on reasonable request.
